# Persistent Infection and Promiscuous Recombination of Multiple Genotypes of an RNA Virus within a Single Host Generate Extensive Diversity

**DOI:** 10.1371/journal.pone.0000917

**Published:** 2007-09-19

**Authors:** Ziming Weng, Roger Barthelson, Siddarame Gowda, Mark E. Hilf, William O. Dawson, David W. Galbraith, Zhongguo Xiong

**Affiliations:** 1 Department of Plant Sciences, University of Arizona, Tucson, Arizona, United States of America; 2 Citrus Research and Education Center, University of Florida, Lake Alfred, Florida, United States of America; 3 United States Department of Agriculture-Agricultural Research Service-United States Horticulture Research Laboratory, Fort Pierce, Florida, United States of America; The Australian National University, Australia

## Abstract

Recombination and reassortment of viral genomes are major processes contributing to the creation of new, emerging viruses. These processes are especially significant in long-term persistent infections where multiple viral genotypes co-replicate in a single host, generating abundant genotypic variants, some of which may possess novel host-colonizing and pathogenicity traits. In some plants, successive vegetative propagation of infected tissues and introduction of new genotypes of a virus by vector transmission allows for viral populations to increase in complexity for hundreds of years allowing co-replication and subsequent recombination of the multiple viral genotypes. Using a resequencing microarray, we examined a persistent infection by a *Citrus tristeza virus* (CTV) complex in citrus, a vegetatively propagated, globally important fruit crop, and found that the complex comprised three major and a number of minor genotypes. Subsequent deep sequencing analysis of the viral population confirmed the presence of the three major CTV genotypes and, in addition, revealed that the minor genotypes consisted of an extraordinarily large number of genetic variants generated by promiscuous recombination between the major genotypes. Further analysis provided evidence that some of the recombinants underwent subsequent divergence, further increasing the genotypic complexity. These data demonstrate that persistent infection of multiple viral genotypes within a host organism is sufficient to drive the large-scale production of viral genetic variants that may evolve into new and emerging viruses.

## Introduction

The emergence of new viruses is a constant challenge to the well-being of the human race and its food supply. New viruses or viral strains are produced from existing forms as a consequence of two processes: mutation and recombination or reassortment, which occur in both plant and animal hosts [1]–[6]. The potential for recombination and reassortment is greatly enhanced in persistent and chronic infections, in which multiple genotypes of a single viral species, or multiple viral species, are introduced into a single host through repeated infections [7], [8]. Co-replicating viral genotypes create an environment conducive for RNA recombination to generate potentially new combinations of genes or protein domains that are exponential to the number of genotypes in the mixed infection. These recombinants may evolve subsequently into new and emerging viruses [1], [4]–[7]. However, the extent to which such long-term infections result in genotypic variants remains largely unexplored.

*Citrus tristeza virus* (CTV) represents an example of a virus that causes persistent infections in a long-lived, economically important hardwood perennial crop plant, so that with time a single host plant may become infected by multiple, phylogenetically distinct CTV genotypes. CTV is a member of the genus *Closterovirus* within the family *Closteroviridae* [9]–[12], and is the most important and destructive virus of citrus [13], [14]. CTV virions are flexuous rods, 2000 nm in length and 12 nm in diameter, consisting of one single-stranded, (+)-sense RNA genome encapsidated by two species of coat proteins (97% CP and 3% CPm) [15]. The 19.2 to 19.3 kb genome contains 12 open reading frames, is the largest of the plant RNA viruses, and is one of the largest of all RNA viruses [9]–[11], [16]–[18]. The 5′ half of the genome (∼nt. 1–11,000) encodes proteins (RNA-dependent RNA polymerase, helicase, methyltransferase, and proteases) that are required for viral replication [19] and are thought to be translated directly from the genomic RNA. The 3′ half encodes proteins which are thought to interact with host plants [20]–[22] and are expressed from ten 3′ co-terminal subgenomic RNAs [23], [24].

The global CTV population is very diverse, with numerous, disparate strains [14], [25], many inducing different types and degrees of disease symptoms on different citrus species and varieties. Often in natural infections in the field, CTV exists as a complex comprising multiple strains or genotypes, due to the longevity of individual citrus trees and the extensive use of vegetative propagation of budwood. Continual vertical transmission coupled with repeated horizontal transmission mediated by aphids throughout the history of citrus cultivation has led to the complexity of the CTV population increasing over hundreds of years, resulting in the co-existence of multiple CTV genotypes in a single host [8], [26], [27]. The presence within a host of multiple replicating CTV genotypes and the relatively long periods of co-replication create opportunities for recombination between the genotypes, leading to extensive viral diversity. In this report, we characterized a persistent infection by multiple CTV genotypes by genome-wide microarray resequencing analysis and deep sequencing analysis of selected genomic regions. Our results demonstrate an extraordinary amount of viral variability generated by promiscuous recombination between multiple genotypes, and provide evidence for subsequent divergence of the recombinants within a single host plant.

## Results

### Resequencing analysis of FS2-2 reveals presence of multiple CTV genotypes

To study the CTV genetic complexity of CTV in detail at the sequence level, we designed and validated an Affymetrix resequencing microarray that queries entire genomes of multiple, phylogenetically distinct CTV genotypes [28]. Sequences tiled on the microarray include full-length sequences of four CTV type strains, T3 (Hilf, unpublished), T30 [9], T36 [10], and VT [11], three-quarters of the strain T68-1 genome (Hilf, unpublished), as well as unique genomic sequences identified from five other CTV isolates [28]. Together, over 117 kb of CTV sequences representing a genetic diversity equivalent to ten full-length CTV genomes (Table 1) were tiled on the resequencing microarray. Using known CTV isolates as the source for target cDNAs, the CTV resequencing microarray yielded call rates of ∼99.7–99.8% and call accuracies of ∼99.9–100% [28], performing comparably to, or better than, several reported resequencing microarrays [29], [30].

**Table 1:** Numbers of nucleotides tiled on the CTV resequencing microarray from the indicated source isolates.

CTV isolates	No. of Bases
T30	19,259
T36	19,293
VT	19,226
T3	19,253
T68-1*	13,585
SY568 unique sequence (5′ VT+3′ T30)**	8,090
H33 unique sequence (VT)	8,391
NUagA unique sequence (VT)	9,991
T385 unique sequence (T30)	127
Qaha unique sequence (T36)	2,298
**Total nucleotides**	**117,088**
Internal control nucleotides	807
**Total Probes**	**943,160**

* The genomic sequence of T68-1 is incomplete with a contig of 3.7 kb near the 5′ end and a contig of 9.6 kb at the 3′ end.

** Isolate within the parenthesis is the completely tiled, representative isolate of the phylogenetic group with which the pairwise comparison was made. 5′, the 5′ half of the genome, 3′, the 3′ half of the genome.

**Figure 1:**
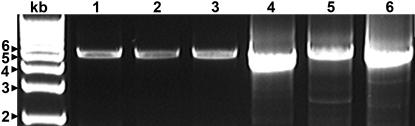
DNA fragments representing the CTV genome amplified by RT-PCR. Total RNA prepared from FS2-2-infected sweet orange leaves was used as the template for RT-PCR. Lanes 1-3, DNA amplified by 3′ primer CTV5427R and three 5′ primers: CTV5endFT36, CTV5endFVT, and FT30CTV5end, respectively; lane 4, DNA amplified by primers CTV5403F and CTV09997R; lane 5, DNA amplified by primers CTV09262F and CTV14630R; and lane 6, DNA amplified by primers CTV14469F and CTV19395R.

We subsequently employed the microarray for analysis of the genetic complexity in a natural CTV isolate, FS2-2. The isolate, collected originally from a citrus grove in Florida, was associated with an unusual stem-pitting symptom in the Hamlin sweet orange. The isolate was initially suspected to possess multiple CTV genotypes on the basis of PCR-amplification with genotype-specific primers (data not shown). The multiple CTV genomes present in FS2-2 were amplified in their entirety by long-range RT-PCR with a high fidelity polymerase from total RNA prepared from infected tissues, using four sets of universal CTV primers (Table 2) capable of amplifying all known CTV genomes. DNA fragments of expected sizes were amplified from the total RNA extracted from FS2-2-infected tissues (Fig. 1). Furthermore, each of the three unique 5′ end PCR primers, designed specifically for CTV genomes with very different 5′ terminal sequences, successfully amplified DNA fragments of the predicted sizes in RT-PCR (Fig. 1), indicating the presence of multiple genotypes in the FS2-2 CTV complex. The three 5′ DNA fragments and the three DNA fragments amplified from the remaining portion of the CTV genomes were pooled proportionally, and then were processed for microarray hybridization [28].

**Table 2:** Primers for RT-PCR amplification of CTV genome and genomic regions.

Set	Primers		Sequences
**1**	CTV5endFT36	Forward	5′ AATTTCACAAATTCAWCCT 3′
	CTV5endFVT	Forward	5′ AATTTCTCAAATTCACCCGTA 3′
	CTV5endFT30	Forward	5′ AWTTTCGATTCAAATTCACC 3′
	CTV5427R	Reverse	5′ AGTTYGATCCYACTTCCATAG 3′
	CTVR6912	RT	5′ TMGSGCACTRTCRGARGAA 3′
**2**	CTV5403F	Forward	5′ CTCSCTATGGAAGTRGGATC 3′
	CTV09997R	Reverse	5′ GCRAARCCAGCGTTCGTCAT 3′
	CTV10170RVT	RT	5′ GGTACTCGCCTTCCATCC 3′
	CTV10170RT36	RT	5′ GATACTCACCTTCCATCC 3′
**3**	CTV09262F	Forward	5′ CCTACYGAATATAAGGGTAG 3′
	CTV14630R	Reverse	5′ TTAACACCACAGGCAACACT 3′
	CTV14887R	RT	5′ ACAAACATMCCTGCCCAAC 3′
**4**	CTV14469F	Forward	5′ TRGCGTCRCT TGGTTTGTTT 3′
	CTV19395R	Reverse	5′ CTACGAAGCTTGGACCTATGT 3′
	CTV3endR	RT	5′ TGGACCTATGTTGGCCCCCC 3′
5′ end1kb	CTV942R	RT/Reverse	5′ AACCGTCCYCGTACGGTTTC 3′
p33	CTV10834F	Forward	5′ GATGTTTGCYTTCGCGAGCG 3′
	CTV11815R	Reverse	5′ AMCCCGTTTAAACAGAGTC 3′
	CTV12124R	RT	5′ AACCCARAAGYACCATACCG 3′

**Figure 2:**
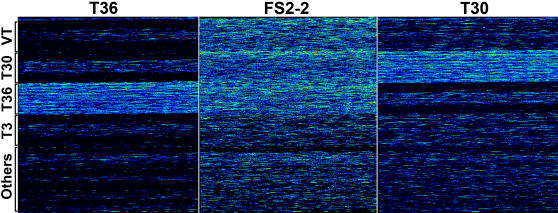
Images of hybridized resequence microarrays. The Affymetrix CTV Microarray chips were hybridized with the target DNA indicated at the top of each panel. Hybridized microarrays were scanned at a resolution of 1.5 µm per pixel. Warm colors represent higher hybridization intensities and cool colors represent lower hybridization intensities. Locations of CTV genomes tiled on the microarray are indicated to the left. Single genome blocks are hybridized with T36 and T30 target DNA while multiple genome blocks are hybridized with FS2-2 target DNA.

The existence of multiple CTV genotypes in the FS2-2 complex was clearly evident in the scanned images of the hybridized resequencing microarray (Fig. 2). Hybridization with target DNA prepared from a single genotype generated a single block of intensive hybridization corresponding to the location on the microarray of the tiling path for that genome (e.g. hybridizations with the T30 and the T36 target DNA in Fig. 2). In comparison, hybridization with the FS2-2 target DNA yielded strong signals in multiple microarray blocks corresponding to the tiled genomes of VT, T30, and T36, indicating that the FS2-2 isolate contained at least three genotypes.

**Figure 3:**
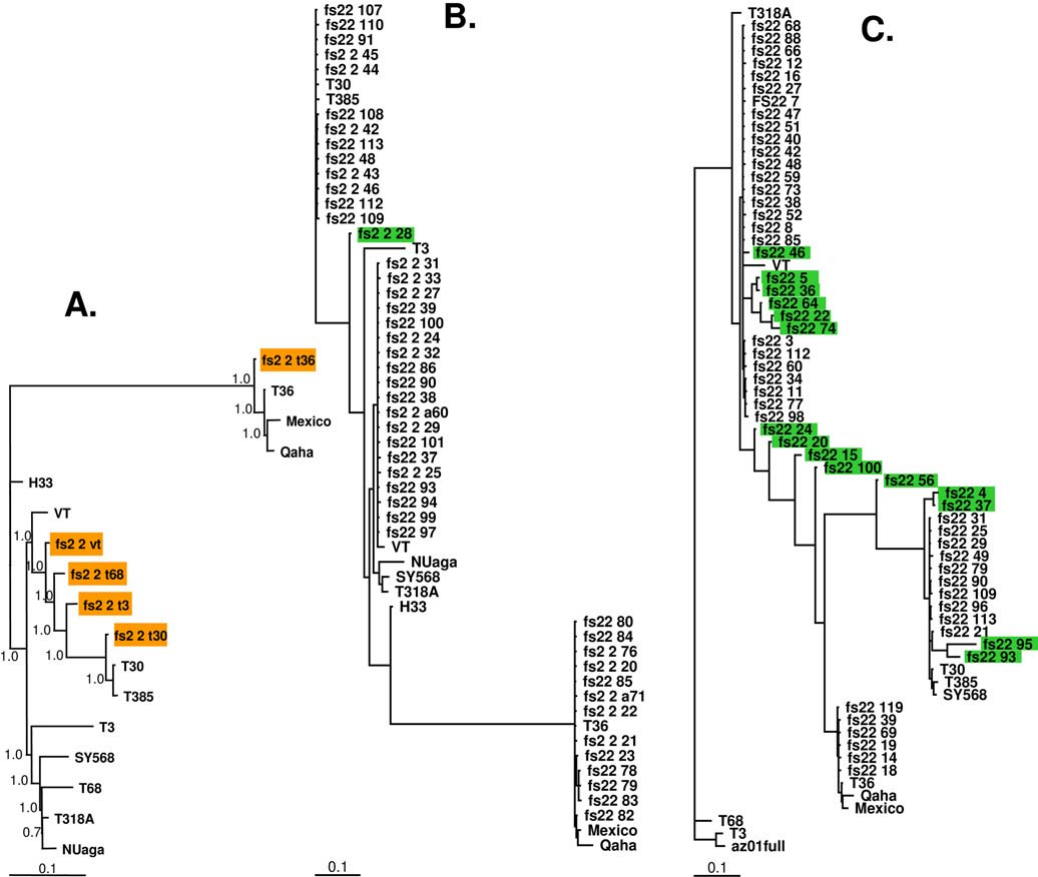
Bayesian phylogenetic inference of CTV genomes and genome fragments. Unrooted, consensus phylogenetic trees were obtained from 2,000,000 generations of the Markov chain Monte Carlo simulation in Bayesian analysis using a general time-reversal model of nucleotide substitution [33]. The number above each branch indicates the Bayesian posterior probability. The scale bars represent 0.1 expected substitutions per site. Branch lengths are proportional to evolutionary distance. Sequences were aligned using ClustalX [47] and subsequently manually aligned prior to the Bayesian phylogenetic analysis. A, Known CTV genomes and CTV genomes assembled from resequencing analysis of FS2-2 (highlighted orange). The suffix at the end of fs2_2 distinguishes multiple genotypes in the isolate and also indicates the anchor sequence from which the consensus contig was generated by the Phrap program. B, the 5′ proximal 1 kb, and C, p33-coding region of CTV genomes obtained by direct sequencing of RT-PCR clones. In both B and C, Bayesian posterior probability and clones with identical sequences were omitted for clarity. Recombinant sequences are highlighted in green.

Hybridization intensities of each probe on the resequencing microarray were subsequently processed using the Affymetrix GeneChip Operating Software version 1.4. Base calls were made using the ABACUS algorithm [31] with a haploid model as implemented in the Affymetrix GeneChip Sequence Analysis Software (GSEQ) version 4.0. A total of 252 sequence fragments corresponding to full length CTV genomes and genomic fragments tiled on the microarray were produced by the resequencing analysis. These fragments and the quality scores associated with each base call were used in contig assembly by the Phrap program [32] as implemented in the CodonCode Aligner program. Three consensus contigs of complete CTV genomes (fs2_2_vt, fs2_2_t30, and fs2_2_t36), corresponding to the three visually identified CTV genotypes, were assembled (Fig. 3a). In addition, two partial consensus contigs were also assembled (fs2_2_t3 and fs2_2_t68). Both were placed as intermediates between fs2_2_vt and fs2_2_t30 by Bayesian phylogenetic analysis [33] (Fig. 3a), and therefore might represent minor components of the CTV complex or variants generated by recombination between the major genotypes.

### Deep sequencing analysis of the 5′ 1 kb of isolate FS2-2 confirms existence of multiple genotypes

To verify the presence of multiple CTV genotypes in the FS2-2 complex, a fragment of approximately 1 kb in size, corresponding to the 5′ termini of the CTV genomes, was amplified by RT-PCR and cloned. The 5′ terminal region was targeted because of its unusually high sequence variability in the CTV genomes. Previous comparative sequence analysis revealed an interesting and unusual distribution of sequence variation across the CTV genome [11], [12], [16], [34]. The 3′ halves of the CTV genomes were highly conserved, with sequence identity being at or above 90%, as would be expected of strains of the same virus. However, sequences of the 5′ halves were much more divergent, with pairwise comparisons yielding sequence identities of 70% or less for some isolates. Sequence identities as low as 48% was observed at the 5′ untranslated regions (UTRs) [9], [35]. The 5′ half of the CTV genome encodes a polyprotein that contains motifs characteristic of RNA-dependent RNA polymerases, helicases, methyltransferases, and proteases, and is required for viral replication [19]. Generally speaking, viral proteins required for replication are highly conserved within viral species and genera, and occasionally this conservation may extend across an entire family of viruses.

The 5′ genomic fragment of CTV targeted for RT-PCR amplification includes the highly divergent 5′ UTRs of approximately 100 nucleotides, and approximately 830 nucleotides from the 5′ open reading frame that encodes for the polyprotein required for CTV replication. RT-PCR amplification of the fragment was accomplished using a combination of three 5′ end primers and a universal, 3′ primer (Table 2) that were designed for the amplification of all known CTV sequences at the 5′ end. A total of 70 clones were randomly selected and sequenced using an ABI 3730XL DNA Analyzer. Among the clones sequenced in this manner, 15 contained sequences nearly identical (98.7∼99.8% identity) to the reported T36 sequence [10]. Sequences of 20 clones were nearly identical (99.3∼99.6% identity) to the published T30 sequence [9], while another 34 clones produced sequences highly similar (97.6∼97.9% identity) to that of VT [11]. These results unequivocally identified three CTV genotypes in the FS2-2 complex that corresponded to the three major genotypes revealed by the resequencing microarray. Within each of the T30-like and the VT-like genotypes, the clones were nearly homogeneous, with a sequence identity of 99.3∼100%. However, sequences within the T36-like genotype were found to be slightly more diverse, with a sequence identity ranging from 98.6% to 100%, suggesting that this genotype might have evolved faster and started to diversify within the population. Indeed, Bayesian phylogenetic analysis showed that three sequences (fs22_78, fs22_79, and fs22_83) of the T36-like genotype emerged as a distinguishable subclade within the T36-like clade (Fig. 3b).

Different degrees of divergence were also evident in the number of identical sequences obtained from clones of each of the three major genotypes. Nearly one half (15) of the clones from the VT-like genotype were completely identical, indicating that genomes of the VT-like genotype were quite uniform with little divergence. In contrast, only three of the 15 sequenced clones of the T36-like genotype contained the same sequence, suggesting the genotype has diverged more significantly. Sequence divergence of the T30-like genotype appeared to be between those of the T36- and VT-like genotypes. Among the 20 T30-like clones sequenced, one group of four clones and another group of five clones produced identical sequences with a single nucleotide difference between the groups.

More interestingly, one sequenced clone, fs22_28, occupied an odd place between the T30-like clade and the VT-like clade in the phylogenetic tree (Fig. 3b). This placement was similar to that of the two minor genotypes (fs2_2_t3 and fs2_2_t68) identified by the resequencing analysis (Fig. 3a). Therefore, fs22_28 likely represented one of the minor genotypes identified in the resequencing analysis. Further analysis revealed fs22-28 to be recombinant: its 5′ region comprised 282 nucleotides identical to the sequences of 7 independently sequenced clones from the T30-like genotype, and its 3′ region consisted of 574 nucleotides identical to sequences in 22 sequenced clones of the VT-like genotype. The recombination crossover site in fs22_28 comprised 41 nucleotides identical to sequences within both parental genotypes. Although the recombinant clone represents one of the minor genotypes detected by the resequencing microarray, direct cloning and sequencing of the 5′ 1 kb of the CTV genomes in the FS2-2 complex failed to identify all the minor genotypes.

### CTV p33 ORF exhibits promiscuous genotypic recombination

To search for additional CTV genotypes identified by resequencing analysis in the FS2-2 complex, an additional 1 kb region containing the entire p33 ORF was selected for further RT-PCR cloning and deep sequencing analysis. The p33 ORF of CTV has no homologue in other closteroviruses [36]. The protein it encodes has been shown to be required for CTV movement in specific hosts (Dawson, unpublished data). The p33 ORF, located about 11 kb from the 5′ end of the genome, is more conserved among CTV isolates than is the 5′ end 1 kb region, yet it retains significant sequence divergence for differentiation of strains and isolates. The higher sequence conservation made it possible to design a set of universal RT-PCR primers capable of amplifying all known CTV genotypes non-selectively (Table 2).

A total of 84 RT-PCR clones derived from the p33 ORF of isolate FS2-2 were chosen randomly for deep sequencing analysis. Sequencing data from the 983 nucleotides of these clones (Fig. 3c) provided a detailed portrait of the CTV complex, and of on-going, promiscuous recombination among various major CTV genotypes. Among the sequenced clones, the three major genotypes again were well represented with 51 VT-like clones, twelve T30-like clones, and six T36-like clones. Several groups of clones shared identical sequences (groups of nine, seven, five, five, two, two, and two clones for the VT-like genotype; and groups of two and two clones for the T30-like genotype) while all T36-like clones had unique sequences. These confirmed that the VT-like genotypes were more homogeneous and the T36-like genotypes were more divergent in FS2-2, an observation arising earlier from analysis of the 5′ terminal CTV fragments. The large number of identical sequences obtained from these clones, as well as from clones derived from the 5′ 1 kb region, also demonstrated that the RT-PCR employed in this study maintained a high fidelity during viral sequence amplification and that errors introduced during RT-PCR were negligible.

**Figure 4:**
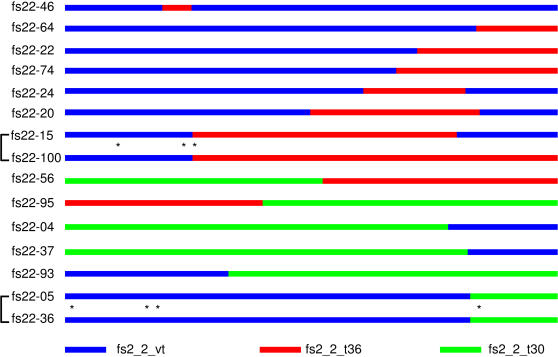
Schematic of CTV recombinants in the p33 ORF. The different parental sequences are represented by specific colored lines; blue (VT), red (T36) and green (T30). Recombinants with identical crossover sites are connected by brackets. Asterisks between the paired recombinants denote locations of diverged nucleotides.

A surprisingly large proportion of the clones (15 clones, 17.9%) were recombinants (Fig. 4). Eight were recombinants between fs2_2_vt and fs2_2_t36, five were recombinants between fs2_2_t30 and fs2_2_vt, and two were recombinants between fs2_2_t30 and fs2_2_t36. Further, four recombinants contained two crossovers, resulting from either a double-crossover or two independent recombination events. The parental sequences for each recombinant were readily identified among the three major genotypes. The crossover sites appeared throughout the p33 ORF without an apparent recombination hotspot. One common feature of these recombinants was that recombination crossover sites contained a stretch of sequence that was identical in both parental molecules. The identical sequences at the crossover sites varied in length, ranging from as short as 3 nucleotides to as long as 25 nucleotides. This feature of shared parental sequences suggested that the recombinants were most likely formed by a template-switching event during viral RNA replication [37]–[39]. Some of the crossover sites were characteristic of classical recombination hotspots with a stretch of AU-rich sequence that promotes dissociation and switching of RNA polymerase from one template to another [40], [41]. However, others lacked such sequences.

These recombinants represented a remarkable amount of genetic variability (Fig. 3c). A large number of recombinants were placed as intermediates between the VT clade and the T30 clade by Bayesian analysis. These recombinants could represent the minor genotypes detected by the resequencing microarray. Since the resequencing microarray is not sensitive enough to differentiate individual recombinants, the minor genotypes identified by the resequencing analysis could very well represent composites of these recombinant genomes. Although the crossover sites were scattered throughout the p33 coding region, all but one of the recombinants maintained the correct reading frame, suggesting that the recombinant p33 protein was also expressed. Therefore, this genetic diversity was expressed at both nucleotide and protein levels.

To eliminate the possibility that the recombinant clones were artificially generated by PCR amplification from a pool of DNA template representing three different genotypes, a control PCR amplification was carried out. Equimolar amounts of two cloned DNA molecules, representing the p33 ORF cloned from the VT-like genotype and from the T36-like genotype respectively, were combined to mimic a mixture of different genotypes, and this was used as the template for the control PCR amplification with the same set of PCR primers and identical PCR conditions. The DNA fragments amplified from this artificial mixture were cloned, and 75 random clones were sequenced. Sequence analysis showed that 26 clones were derived from the T36-like genotype and 49 were derived from the VT genotype. More importantly, none of the 75 sequenced clones was recombinant between the two genotypes present in the template DNA. This result strongly suggests that the extraordinarily large number of recombinant viral molecules detected in the FS2-2 CTV complex represent natural recombinants. Although generation of artificial recombinant during PCR has been reported under specific conditions [42], our results clearly showed that the template DNA molecules and the PCR conditions used in these experiments did not favor the formation of artificial recombinants.

Further analysis of the recombinants shed light on the emergence of new CTV genotypes through recombination and subsequent divergent evolution. This was particularly evident in two pairs of recombinants (Fig. 4). The first pair (fs22-05 and fs22-36) contained an identical recombination crossover site but four divergent nucleotides located both 5′ and 3′ of the crossover site. A plausible explanation is that they originated from a single recombination event and that progenies of the recombinant diverged subsequently. The second pair of recombinants (fs22-15 and fs22-100) contained an identical crossover site at the 5′ end, but fs22-15 possessed an additional crossover site toward the 3′ end. One probable explanation is that both were the progeny of a single recombination event at the 5′ end with fs22-15 subsequently undergoing a further round of recombination. In the identical region of the two recombinants, three divergent nucleotides were found spanning the 5′ crossover site, indicating that the regions had diverged after the first recombination event. These data suggest an event line of recombination, divergence, and further recombination, leading to a distinct CTV genotype. This scenario also explains the chimeric nature of the sequenced SY568 CTV genome [12] as a consequence of recombination between two CTV genotypes [8].

## Discussion

The complexity of a natural CTV isolate consisting of multiple genotypes was revealed through a combination of genome-wide resequencing analysis using a CTV Affymetrix GeneChip resequencing microarray and deep sequencing of selected genomic regions in this study. Furthermore, deep sequencing analysis illustrated an unparalleled level of promiscuous recombination between multiple, co-replicating genotypes in a persistent infection.

Viruses have been a favorite target for resequencing analysis using oligonucleotide microarrays because of their small genome sizes [29], [43]–[46]. However, most of these applications either targeted viruses with relatively little sequence variability, such as severe acute respiratory syndrome virus [29], or only selected regions of viruses having larger sequence variability [43]–[45]. With increased tiling capacity, the Affymetrix resequencing microarrays can now be used to simultaneously detect divergent viruses, as we have demonstrated in our work, where three major genotypes and additional minor genotypes of CTV were successfully identified from isolate FS2-2 by resequencing analysis. The three major CTV genotypes investigated by the resequencing analysis were quite divergent with genome-wide nucleotide differences of 11% between the VT- and the T30-like genotypes, 20% between the VT- and the T36-like genotypes, and 19% between the T30- and the T36-like genotypes. Successful resequencing analysis of these divergent CTV genomes with a single resequencing microarray chip is largely attributed to the tiling strategy of selecting representative full-length CTV genomes guided by phylogenetic analysis and of selecting unique sequences identified by pair-wise comparisons from other CTV genomes. This strategy allows the CTV resequencing microarray with a tiling capacity of 117 kb to encompass a sequence diversity equivalent to ten full-length CTV genomes. With its ability to query concurrently entire genomes of multiple CTV genotypes as demonstrated in this work, the CTV resequencing microarray will find many uses in future CTV studies.

CTV has been known to exist as a complex in nature [8], [25]–[27] likely because of the longevity of the host plant, continuous vertical viral transmission by grafting, and repeated horizontal viral transmission by aphids. In previous studies, characterization of CTV complexes was limited to analysis of genotype-specific PCR markers [25], [27] or to sequencing analysis of selected regions representing a small percentage of the CTV genome [8], [26]. In this study, a genome-wide approach was used to characterize multiple genotypes in a CTV complex for the first time. The availability of complete genomic sequences of co-replicating and interacting CTV genotypes should facilitate detailed and sophisticated analyses of genotypic interactions in any given CTV complex in future studies.

The presence of multiple, co-replicating CTV genotypes was expected to promote recombination. However, the extraordinarily large proportion of recombinant viral molecules as a consequence of promiscuous recombination in the FS2-2 isolate was surprising. We found that 17.6% of cloned and sequenced molecules from the p33 ORF were recombinants derived from the three predominant genotypes in the FS2-2 complex. In contrast, a previous study of another CTV complex, SY568, identified less than 4% of the cloned molecules as being recombinants between two genotypes [8]. It is not clear whether the large proportion of recombinants in FS2-2 is a consequence of an elevated recombination rate in the presence of more than two genotypes or a result of recombinants accumulating over time. Likely though, as a result of current horticultural practices, the sweet orange tree from which FS2-2 was derived was planted free of CTV and the CTV population is a result of ingress of the T30, T36 and VT genotypes by aphid transmission, followed by recombination to produce the recombinants found in this study. Subsequent transmission of the FS2-2 complex by aphids or by vegetative propagation to a similar or a new host may perpetuate the same complexity or alter it by generating new and additional recombinant genotypes.

There was a substantial difference in the proportions of the recombinant sequences obtained upon cloning the 5′ 1 kb (one out of 70 clones) and the p33 ORF (15 out of 84 clones), suggesting that the sequence most proximal to the 5′ end of the CTV genome was more recalcitrant to recombination or that the terminal 1000 bases are subject to more stringent purifying selection, due perhaps to decreased fitness of recombinants, so consequently fewer recombinants are recovered. This difference perhaps can explain the unusual distribution of sequence variability in the CTV genome, with the 5′ half being more variable and the 3′ half being less variable.

Recombination in RNA viruses has been extensively documented [1], [4] as a powerful driving force for generating new sequences which appear as emerging viruses, since recombination can rapidly generate new genotypes by swapping genes or protein domains to reconstitute proteins with novel host-colonizing and pathogenicity traits [5], [6]. The unprecedented scope of recombination between multiple CTV genotypes within a single, persistently infected host as revealed in this study further underscores the importance of recombination in RNA virus evolution, and may explain the extraordinary diversity observed in CTV today. The large number of genetic variants generated through recombination can potentially evolve on their own and become an emerging viral isolate when, and if transmitted to a new host or into a new environment. The influenza virus responsible for the 1918 Spanish flu pandemic was hypothesized to have acquired, through recombination, a portion of the hemagglutinin gene, a key virulence gene, from a swine-lineage influenza [5]. In this regard, it is interesting to note that all but one of the recombinants in this study maintained the correct open reading frames, and consequently generating a functional recombinant protein. Further studies are needed to determine the viability of these CTV recombinants in isolation from the parental sequences and to evaluate the likelihood that any of the recombinants will emerge as a new CTV strain. CTV shares similarities in its genome organization and gene expression strategies with the largest animal RNA viruses, corornaviruses (which includes the viral agent of SARS), so it is conceivable that similar processes may also operate in global populations of coronaviruses to generate genetic diversity.

## Materials and Methods

(See Supporting Information Text S1 for experimental details)

### CTV isolates and genomic sequences

CTV isolate FS2-2 was collected from a citrus grove in Florida in 2004 and maintained on Madam Vinous sweet orange in an insect-proof greenhouse. Full-length genomic sequences of CTV isolates NUagA, Qaha, SY568, T30, T36, T385, and VT were retrieved from GenBank. In addition, full-length unpublished sequences of CTV isolates T3 (M.E. Hilf, unpublished) and H33 (T.E. Mirkov, personal communication) and a partial sequence (13,585 nt) of the CTV T68-1 isolate (M.E. Hilf, unpublished) were included.

### Amplification of the CTV genome by RT-PCR

Full-length genomic equivalents of CTV from each sample were amplified from each sample as four DNA fragments ranging from 4.5 to 5.5 kb by RT-PCR, using four sets of RT-PCR primers (Table 2). Total RNA was extracted from CTV-infected tissue using the Trizol reagent (InVitrogen, Carlsbad, CA). Reverse transcription was carried out using the ImProm II reverse transcriptase (Promega, Madison, WI). CTV genomic fragments were then amplified by 35 cycles of long range PCR using the Stratagene EXL DNA polymerase (Stratagene, La Jolla, CA).

### RT-PCR amplification, cloning, and sequencing of CTV genome fragments

The 5′ 1 kb fragments of the CTV genome were amplified using the three different 5′ PCR primers and a 3′ conserved universal primer (Table 2). The 1 kb fragments containing the p33 ORF were amplified using a set of universal RT-PCR primers (Table 2). PCR products were purified using the Qiagen MinElute PCR Purification Kit (Qiagen, Valencia, CA) and were employed directly for TA-cloning using a pUC18-based vector containing twin *Xcm* I restriction sites^25^. Plasmid DNA from randomly selected clones was purified and sequenced in both directions using an ABI 3730XL DNA Analyzer.

### Microarray hybridization and base-calling

Equimolar amounts of each PCR fragment were pooled, and labeled with biotin-dNTP by terminal deoxynucleotidyl transferase. Hybridization of the labeled target DNA to the microarrays, washing, and subsequent staining in GeneChip Fluidics Station 450 were performed in strict accordance with the instructions provided by Affymetrix. The stained microarray was then scanned at a resolution of 1.563 µm/pixel using a GeneChip Scanner 3000 (Affymetrix, Santa Clara, CA). The final probe intensity data were analyzed with the Affymetrix GeneChip Sequence Analysis Software (GSEQ) to extract sequencing information. Base calls were made using the ABACUS (adaptive background genotype calling scheme) algorithm [31].

### Contig assembly of sequence fragments generated by resequencing analysis

Sequence fragments and the associated quality scores generated by GDAS were converted into fasta-format files and used to assemble full and partial CTV genomic contigs using the Phrap program [32] implemented in the CodonCode Aligner (CodonCode, Dedham, MA).

### Sequence analysis

Sequences of CTV genomes or genomic fragments were aligned using the default parameters of the ClustalX program [47]. Bayesian inference of phylogenetic relationships was carried out using the general time reversal model with gamma-shaped rate variation and a proportion of invariable sites (GTR+I+G) as implemented in MrBayes 3.12 [33]. Phylogenetic trees were then visualized using the TreeView program [48]. Recombinant molecules and their cross-over junctions were determined by RDP2, a recombination detection program that deploys 10 published methods to detect recombinant sequences and recombination breakpoints [49].

## Supporting Information

Text S1 Online Supplemental Materials and Methods for Persistent infection and promiscuous recombination of multiple genotypes of an RNA virus within a single host generate extensive diversity
(0.05 MB PDF)
