# Supplementary material for: Persistent Infection and Promiscuous Recombination of Multiple Genotypes of an RNA Virus within a Single Host Generate Extensive Diversity
Source: PLoS One. 2007 Sep 19;2(9):e917. doi: 10.1371/journal.pone.0000917 (PMC1975466; doi:10.1371/journal.pone.0000917)
Supplement: Text S1 — Online Supplemental Materials and Methods for Persistent infection and promiscuous recombination of multiple genotypes of an RNA virus within a single host generate extensive diversity (0.05 MB PDF) [file pone.0000917.s001.pdf]

Online Supplemental Materials and Methods for

Persistent infection and promiscuous recombination of multiple genotypes of an RNA virus within a single host generate extensive diversity

Viral isolates and genomic sequences

CTV isolate FS2-2 was collected from a citrus grove in Florida in 2004, and was maintained on Madam Vinous sweet orange in an insect-proof greenhouse. The isolate was associated with an unusual stem-pitting symptom in a Hamlin sweet orange tree in the field. Full-length genomic sequences of seven CTV isolates were retrieved from GenBank: T30, a mild isolate from Florida [1]; T36, a decline-inducing isolate from Florida [2]; SY568, a decline and sweet orange stem pitting isolate from California [3]; VT, a decline and seedling yellows isolate from Israel [4]; T385, an essentially symptomless isolate from Spain which is nearly identical to isolate T30 [5]; NUagA, a seedling yellows isolate from Japan (Accession No. AB046398); and Qaha, a T36-like isolate from Egypt (Accession No. AY340974). In addition, full-length sequences were included of CTV isolate T3 (unpublished) from Florida and H33 from Texas (T. E. Mirkov, personal communication) and a partial sequence (13,585 nt) of the CTV T68 isolate from Florida (unpublished).

Amplification of CTV genome by RT-PCR

Complete full-length genomes of CTV from isolate FS2-2 were amplified as four DNA fragments by reverse transcription-polymerase chain reaction (RT-PCR), using four sets of primers. Each set consisted of an RT primer and a pair of PCR primers, and were designed using sequences highly conserved in all known CTV genomes. The extreme sequence diversity at the 5' ends of CTV genomes necessitated three separate primers, one each for the T36-, VT-, and

T30-like CTV genomes. Together, the four sets of primers were capable of amplifying all known CTV genomes as four DNA fragments ranging from 4.5 to 5.5 kb. Total RNA was extracted from a sample (1 g) of CTV-infected tissue using the Trizol reagent (Invitrogen, Carlsbad, CA) as described by the manufacturer. Reverse transcription was carried out at 42°C for 90 minutes using the ImProm II reverse transcriptase (Promega, Madison, WI) with the protocol provided by the manufacturer. CTV genomic fragments were then amplified by 35 cycles of long range PCR using the high fidelity Stratagene EXL DNA polymerase (Stratagene, La Jolla, CA). Each cycle of PCR consisted of a 30-second denaturation at 92°C, a 30-second annealing at 50°C, and a 5-minute polymerization at 68°C, with a 2-second increment after each cycle. The PCR program began with an initial 2-minute denaturation at 92°C, and terminated with a final 10-minute polymerization at 68°C.

RT-PCR amplification, cloning, and sequencing of CTV genome fragments

The 5' fragments (1 kb) of the CTV genomes were amplified using the three 5' PCR primers and a 3' conserved universal primer, CTV942R, complementary to a highly conserved sequence located approximately 1 kb downstream from the 5' end . CTV942R was used as both the RT and the PCR primer. The 1 kb fragments containing the p33 ORF near the middle of CTV genome were amplified using a set of universal RT-PCR primers capable of amplifying all known CTV genotypes non-selectively. The set comprised an RT primer, CTV12124R, complementary to nucleotides 12124 to 12105, a 5' PCR primer, CTV10834F, identical to nucleotides 10834 to 10853, and a 3' PCR primer, CTV11815R, complementary to nucleotides 11815 to 11794 of the aligned CTV genomes. Reverse transcription of CTV genomic cDNA was carried out using the ImProm II reverse transcriptase at 42°C for 1 hour and amplification of the DNA fragments was carried out by 35 cycles of PCR using Taq DNA polymerase (Promega, Madison, WI). Each

PCR cycle contained a 45-second denaturation at 94°C, a 1-minute annealing at 46°C, and a 1-minute polymerization at 72°C with a 2-second increment after each cycle. A 2-minute denaturation at 94°C was programmed at the beginning and a 10-minute polymerization at 72°C at the end.

PCR products were purified using the Qiagen MinElute PCR Purification Kit (Qiagen, Valencia, CA), and were employed for TA-cloning using a pUC18-based vector containing twin *Xcm* I restriction sites [6]. Clones with inserts were identified by colony-PCR screening using the M13F and M13R primers that annealed to sequences upstream and downstream of the multiple cloning sites in the vector. Plasmid DNA molecules from randomly selected clones were purified and sequenced in both directions using an ABI 3730XL DNA Analyzer at the Genomic Analysis and Technology Core Facility at the University of Arizona.

Design of CTV resequencing microarray

Genomic sequences of representative CTV isolates were selected for microarray tiling based on the tiling capacity of the resequencing microarray. Nine full-length CTV genomes (T30, T36, VT, SY568, T385, NUagA, Qaha, T3, and H33) available at the time were phylogenetically analyzed using the ClustalX program [7]. The T68 genomic sequence of 13.3 kb was incomplete, and therefore was not included in this analysis. Four apparent clades of CTV isolates, identified by this and other analyses [8,9], guided the selection of a representative genomic sequence from each clade: T36 representing the clade of T36 and Qaha isolates; T30 representing the clade of T30, T385, and SY568 isolates; VT representing the clade containing VT, NUagA, and H33 isolates; and T3 representing the singleton T3 clade. Full-length genomic sequences of T36, T30, VT, and T3 were fully tiled on the resequencing microarray. Although T68 was not included in the original phylogenetic analysis, subsequent analysis of the 5' 3.5 kb sequence of T68 with

those of other isolates clearly established T68 as being uniquely different from the four CTV clades. Consequently, all 13,585 available nucleotides of the T68 genome were also completely tiled on the microarray.

Genomes from the remaining CTV isolates in each clade were then compared to that of the tiled, representative isolate to identify unique sequences of the genomes for additional microarray tiling. A unique sequence was defined as a 25 nucleotide region that contained either a run of two or more different nucleotides at the central (13th nucleotide) position, or two or more non-contiguous, different nucleotides within eight nucleotides of the 13th nucleotide. These parameters were dictated by the fact that a run of centrally located, mismatched nucleotides or closely-spaced mismatched nucleotides would significantly destabilize the hybridization between the labeled target DNA and the oligonucleotide probe on the microarray. Unique nucleotide sequences identified by this program and selected full genomic sequences of CTV isolates were then tiled on the GeneChip CustomSeq resequencing array with an 8-μm feature size using the Affymetrix photolithographic manufacturing process (Affymetrix, Santa Clara, CA). The complete tiling of 117,088 nucleotides comprises four full-length CTV genomes, one partial genome, and unique sequences from other CTV isolates. In addition, 807 nucleotides from an artificial cDNA clone were included as an internal control. For each nucleotide tiled on the array, four 25-mer oligonucleotides (a quartet) corresponding to the sense strand and four oligonucleotides representing the antisense strand were tiled on the microarray. Thus, the microarray contains a total of 943,160 25-mer oligonucleotide probes.

Microarray hybridization and base-calling

Amplified PCR fragments were cleaned using the Qiagen MinElute PCR Purification Kit (Qiagen, Valencia, CA), and subsequently quantified using an ND-1000 spectrophotometer

(NanoDrop Technologies, Wilmington, DE). Equimolar amounts of each fragment were pooled; an amount of amplified DNA equivalent to 0.055 pmoles of CTV genome was then fragmented to 20 to 200 bp and labeled with biotin-dNTP by terminal deoxynucleotidyl transferase according to the Resequencing Assay Protocol 2.1 (Affymetrix, Santa Clara, CA).

Hybridization of the target DNA to the microarray was carried out in GeneChip Fluidics Station 450, according to the instructions provided by Affymetrix. The target DNA bound to the probes on the microarray was stained using a three-stage process consisting of streptavidin-phycoerythrin (SAPE) stain, amplification with biotinylated anti-streptavidin antibodies, and a final stain with SAPE. The microarray was then scanned at a resolution of 1.563 µm/pixel using an Affymetrix GeneChip Scanner 3000.

The scanned image was automatically gridded, and the signal for each probe was statistically averaged using the Affymetrix GeneChip Operating Software version 1.4. The final probe intensity dataset was analyzed with Affymetrix GeneChip Sequence Analysis Software (GSEQ) 4.0 to retrieve sequence information. Base calls were made using the ABACUS algorithm [10] with a haploid model. The ABACUS parameters were as follows: no signal threshold = 20, weak signal fold threshold = 20, maximum signal to noise ratio = 20, quality score threshold = 3.0, base reliability threshold across samples = 0, trace threshold = 1, and sequence profile threshold = -0.175.

Contig assembly of sequence fragments generated by resequencing analysis

The output from the GSEQ consisted of 252 sequence fragments, corresponding to the full-length genome and sequence fragments tiled on the microarray. Each nucleotide in these fragments was assigned a quality score by GSEQ, on the basis of differential hybridization of perfect-matches and mismatches in the sense and antisense quartets as well as on the basis of

hybridization characteristics of the neighboring nucleotides. The sequence fragments and the associated quality scores were converted into fasta-format files, and were used to assemble full and partial CTV genomic contigs using the Phrap program [11] implemented in the CodonCode Aligner (CodonCode, Dedham, MA). After experimenting with a wide range of parameters, the following Phrap command parameters produced reliable and reproducible alignments and consensus contigs that reflected the true nature of the genotype(s) in samples: -penalty -2 -minmatch 10 -maxmatch 10 -minscore 12 -vector_bound 0 -masklevel 100 -trim_start 0 -trim_qual 4 -forcelevel 0.

Bayesian phylogenetic and recombination analysis

Sequences of CTV genomes or genomic fragments were initially aligned using the default parameters of the ClustalX program [7], followed by visual inspection and manual alignment as required. Bayesian inference of phylogenetic relationships was carried out using MrBayes 3.12 [12], using the general time-reversal model with gamma-shaped rate variation and a proportion of invariable sites (GTR+I+G). This model was determined as the best-fit model for phylogenetic inferences of the input sequences using the ModelTest program [13]. Two parallel runs of Metropolis-coupled Markov chain Monte Carlo simulation of one cold and three heated chains were then carried out for at least 5,000,000 generations, or until the average standard deviation of split frequencies reached 0.01. One tree was sampled for every 200 generations during the simulation. A consensus tree was constructed from the sampled trees after a burn-in of two-fifth of the sample trees. The Bayesian posterior probability for each node was calculated as the proportion of sampled trees containing the node. With a few exceptions on minor nodes, the posterior probability on all major nodes approached 1.00 after the extensive phylogenetic analysis. Phylogenetic trees were then visualized using the TreeView program [14]. Cross-over

junctions of the recombinant molecules were determined initially by RDP2, a recombination detection program that deploys ten published methods to detect recombinant sequences and recombination breakpoints [15] and subsequently confirmed by visually inspection .

1. Albiach-Marti MR, Mawassi M, Gowda S, Satyanarayana T, Hilf ME, et al. (2000) Sequences of Citrus tristeza virus separated in time and space are essentially identical. Journal of Virology 74: 6856-6865.
2. Karasev AV, Boyko VP, Gowda S, Nikolaeva OV, Hilf ME, et al. (1995) Complete sequence of the Citrus tristeza virus RNA genome. Virology 208: 511-520.
3. Yang ZN, Mathews DM, Dodds JA, Mirkov TE (1999) Molecular characterization of an isolate of citrus tristeza virus that causes severe symptoms in sweet orange. Virus Genes 19: 131-142.
4. Mawassi M, Mietkiewska E, Gofman R, Yang G, BarJoseph M (1996) Unusual sequence relationships between two isolates of citrus tristeza virus. Journal of General Virology 77: 2359-2364.
5. Vives MC, Rubio L, Lopez C, Navas-Castillo J, Albiach-Marti MR, et al. (1999) The complete genome sequence of the major component of a mild citrus tristeza virus isolate. Journal of General Virology 80: 811-816.
6. de Vries E (1998) pUCPCR1 - A vector for direct cloning of PCR products in a double Xcm1 restriction site offering compatible single 3 '-overhanging T residues. Molecular Biotechnology 10: 273-274.
7. Thompson JD, Gibson TJ, Plewniak F, Jeanmougin F, Higgins DG (1997) The CLUSTAL_X windows interface: flexible strategies for multiple sequence alignment aided by quality analysis tools. Nucleic Acids Research 25: 4876-4882.
8. Hilf ME, Mavrodieva VA, Garnsey SM (2005) Genetic marker analysis of a global collection of isolates of Citrus tristeza virus: Characterization and distribution of CTV genotypes and association with symptoms. Phytopathology 95: 909-917.
9. Xiong Z, Barthelson R, Weng Z, Galbraith DW (2006) Designing and testing of a Citrus tristeza virus resequencing microarray. Proceedings of the International Organization of Citrus Virologists 16: 11-22.
10. Cutler DJ, Zwick ME, Carrasquillo MM, Yohn CT, Tobin KP, et al. (2001) High-throughput variation detection and genotyping using microarrays. Genome Research 11: 1913-1925.
11. Ewing B, Hillier L, Wendl MC, Green P (1998) Base-calling of automated sequencer traces using phred. I. Accuracy assessment. Genome Research 8: 175-185.
12. Ronquist F, Huelsenbeck JP (2003) MrBayes 3: Bayesian phylogenetic inference under mixed models. Bioinformatics 19: 1572-1574.
13. Posada D, Crandall KA (1998) MODELTEST: testing the model of DNA substitution. Bioinformatics 14: 817-818.
14. Page RDM (1996) TreeView: An application to display phylogenetic trees on personal computers. Computer Applications in the Biosciences 12: 357-358.
15. Martin DP, Williamson C, Posada D (2005) RDP2: recombination detection and analysis from sequence alignments. Bioinformatics 21: 260-262.
